# Supplementary material for: Oxidative Stress-Induced Sirtuin1 Downregulation Correlates to HIF-1α, GLUT-1, and VEGF-A Upregulation in Th1 Autoimmune Hashimoto’s Thyroiditis
Source: Int J Mol Sci. 2021 Apr 7;22(8):3806. doi: 10.3390/ijms22083806 (PMC8067526; doi:10.3390/ijms22083806)
Supplement: Supplementary file 1 [file ijms-22-03806-s001.pdf]

International Journal of
Molecular Sciences

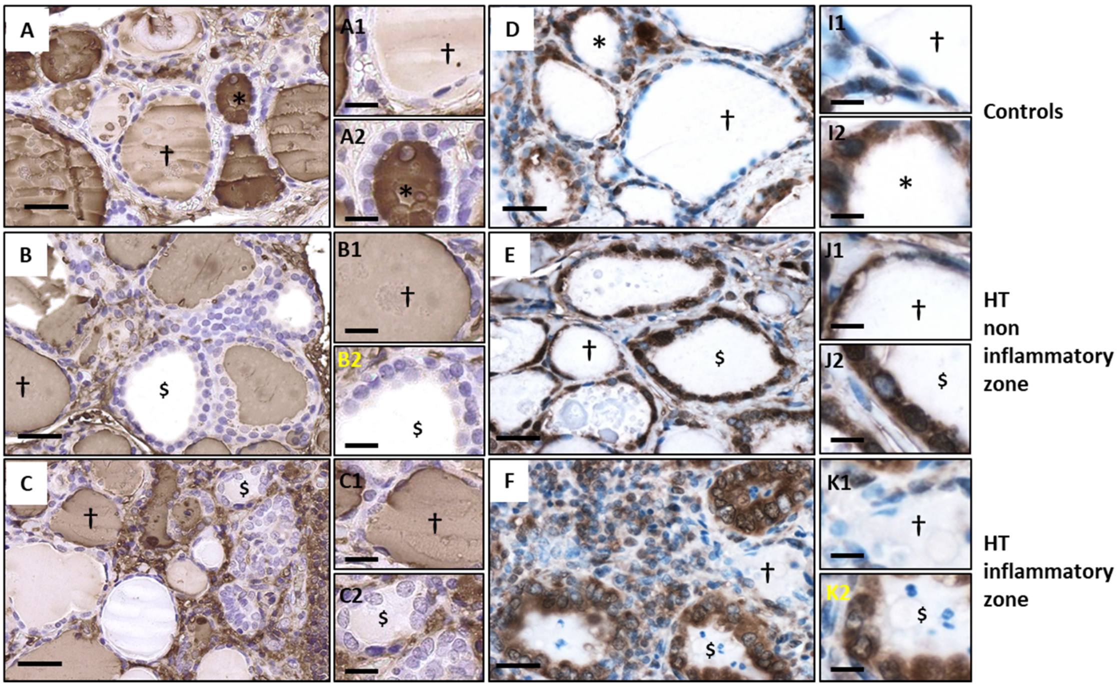

Supplemental Figure 1. Immunohistochemistry on paranodular tissue obtained from multinodular goiter patients designated as controls (A; D) and thyroid tissues from HT patients (B,C,E and F). The pictures illustrate representative tissue samples of both conditions. In control thyroids, T4 was expressed in hypofonctionnal (†) and more intensively in active follicles (*). HNE staining was low in hypofunctionnal and slightly higher in active follicles (*) (D). In HT thyroids, in non-inflammatory and inflammatory zones, T4 was not detected in altered active-like follicles ($) (B-C) which highly expressed HNE (E-F). (C-F) Scale bar = 50µm. (C1-H2) Scale bar = 20µm.
